# Specialized adaptations for springtail predation in Mesozoic beetles

**DOI:** 10.1038/s41598-017-00187-8

**Published:** 2017-03-07

**Authors:** Zi-Wei Yin, Chen-Yang Cai, Di-Ying Huang, Li-Zhen Li

**Affiliations:** 1 Department of Biology, College of Life and Environmental Sciences, Shanghai Normal University, Shanghai, 200234 P. R. China; 2 Key Laboratory of Economic Stratigraphy and Palaeogeography, Nanjing Institute of Geology and Palaeontology, Chinese Academy of Sciences, Nanjing, 210008 P. R. China; 3 State Key Laboratory of Palaeobiology and Stratigraphy, Nanjing Institute of Geology and Palaeontology, Chinese Academy of Sciences, Nanjing, 210008 P. R. China

## Abstract

Insects exhibit a variety of morphological specializations specific to particular behaviors, and these permit the reconstruction of palaeobiological traits. Despite the critical importance of predator-prey strategies in insect evolution, the appearance of particular aspects of predation are often difficult to determine from the fossil record of hexapods. Here we report the discovery of highly specialized, mid-Cretaceous ant-like stone beetles (Staphylinidae: Scydmaeninae) displaying morphological modifications unknown among living scydmaenids and associated with predation on springtails (Collembola), a widespread and abundant group of significantly greater geological age. *Cascomastigus monstrabilis* gen. et sp. nov. exhibits an extremely large body size, elongate clubbed maxillary palpi, toothed mandibles, and more importantly, slender and highly modified antennae that functioned as an antennal setal trap. Such an antennal modification is analogous to that of the modern ground beetle genus *Loricera* (Carabidae: Loricerinae), a group possessing a specialized antennal setal trap exclusively for the capture of springtails. The presence of an identical antennal setal trap in *C. monstrabilis* demonstrates a unique and dramatic form of obligate predation among the late Mesozoic insects.

## Introduction

Predator-prey interactions are a critical component of ecological and evolutionary associations, shaping the success (or lack thereof) of major lineages. Specific associations are often difficult to discern in the fossil record, despite the extensive record of predatory and parasitic taxa in most deposits. This dearth of direct observational evidence for particular predator-prey associations gives the false impression that most extinct species of insect predators were perhaps generalists owing to the absence of data suggesting otherwise. Specialized predator systems are difficult to document in the absence of either extraordinary preservation or unique morphological traits directly correlated with particular biologies.

Springtails (Hexapoda: Collembola), usually only a few millimeters long, are one of the most widespread and abundant of terrestrial arthropods, and is the most diverse group of Entognatha, the sister group to insects. The clade has a global distribution, occurring on every continent, including Antarctica, and species are often found as ‘aerial plankton’^1^. One of the most remarkable and characteristic structures of springtails is their specialized jumping organ (furcula), which has evolved through the basal fusion of a pair of appendages on the fourth abdominal segment and acts like a spring when released from an associated, ventral locking mechanism^1, 2^. Although some have secondarily lost their jumping organs, most springtails are capable of jumping for a long distance, and this leaping has apparently evolved for both dispersion as well as an effective means of predator avoidance. The earliest known Collembola, *Rhyniella praecursor*, from the Early Devonian Rhynie chert (Scotland, approximately 400 million years ago [mya]), is strikingly modern in appearance relative to the surviving family Isotomidae, to which the species likely belongs^3, 4^. These fossils demonstrate that springtails established their specialized mode of locomotion over 400 million years ago^3, 5^. Given their ubiquity and abundance, a wide range of animals are general predators of Collembola^1^, while specialized predators of springtails are limited^6^. Among the known specialized predators are some beetles, wasps, and ants, and in each various body parts or organs are modified for hunting springtails in different manners^6^. Fossil evidence of such specialized predation on springtails, however, has been lacking. The recent discovery of ant-like stone beetles, belonging to the extant staphylinid tribe Mastigini, in mid-Cretaceous amber from Myanmar provides a unique example of a specific and early predatory-prey association, and reveals a group specialized for predation on the abundant resource represented by springtails.

## Results

### Systematic Palaeontology

Order Coleoptera Linnaeus, 1758

Family Staphylinidae Latreille, 1802

Subfamily Scydmaeninae Leach, 1815

Supertribe Mastigitae Fleming, 1821

Tribe Mastigini Fleming, 1821

### *Cascomastigus monstrabilis* Yin & Cai, gen. nov

LSID, urn:lsid:zoobank.org:act:08FE9CB6-4F93-4F68-B397-F2B6D392AF36

### Type species

*Cascomastigus monstrabilis* Yin & Cai, sp. nov. (here designated)

### Diagnosis

*Cascomastigus* are separated from other genera of the Mastigini by the following combination of characters: body size exceptionally large (usually over 6.5 mm); maxillary palpus extremely elongate, with enlarged apical palpomere (palpomere IV) strongly bent basally; and elytron distinctly striate.

### *Cascomastigus monstrabilis* Yin & Cai, sp. nov

LSID, urn:lsid:zoobank.org:act:7D363AB7-A803-4B30-A375-7AF3B46E88CF

### Type material

Holotype, SNUC-Paleo-0005, male, housed in the Insect Collection of the Shanghai Normal University, Shanghai, China. Paratype, NIGP165026, female, housed in the Nanjing Institute of Geology and Palaeontology, CAS, Nanjing, China. Both specimens are derived from the earliest Canomanian amber (approximately 99 mya) from Hukawng Valley, Kachin State, northern Myanmar.

### Diagnosis

As for the genus (see above), with the following minor additions: antenna slightly shorter than body; scape 1.30 (female)–1.62 (male) times as long as head, and 0.96 (female)–1.13 (male) times as long as pronotum; antennomere IV 1.3 (male)–1.8 (female) times as long as III.

### Description

Refer to online Supplemental Information for a complete description and etymology.

## Discussion

**Figure 1:**
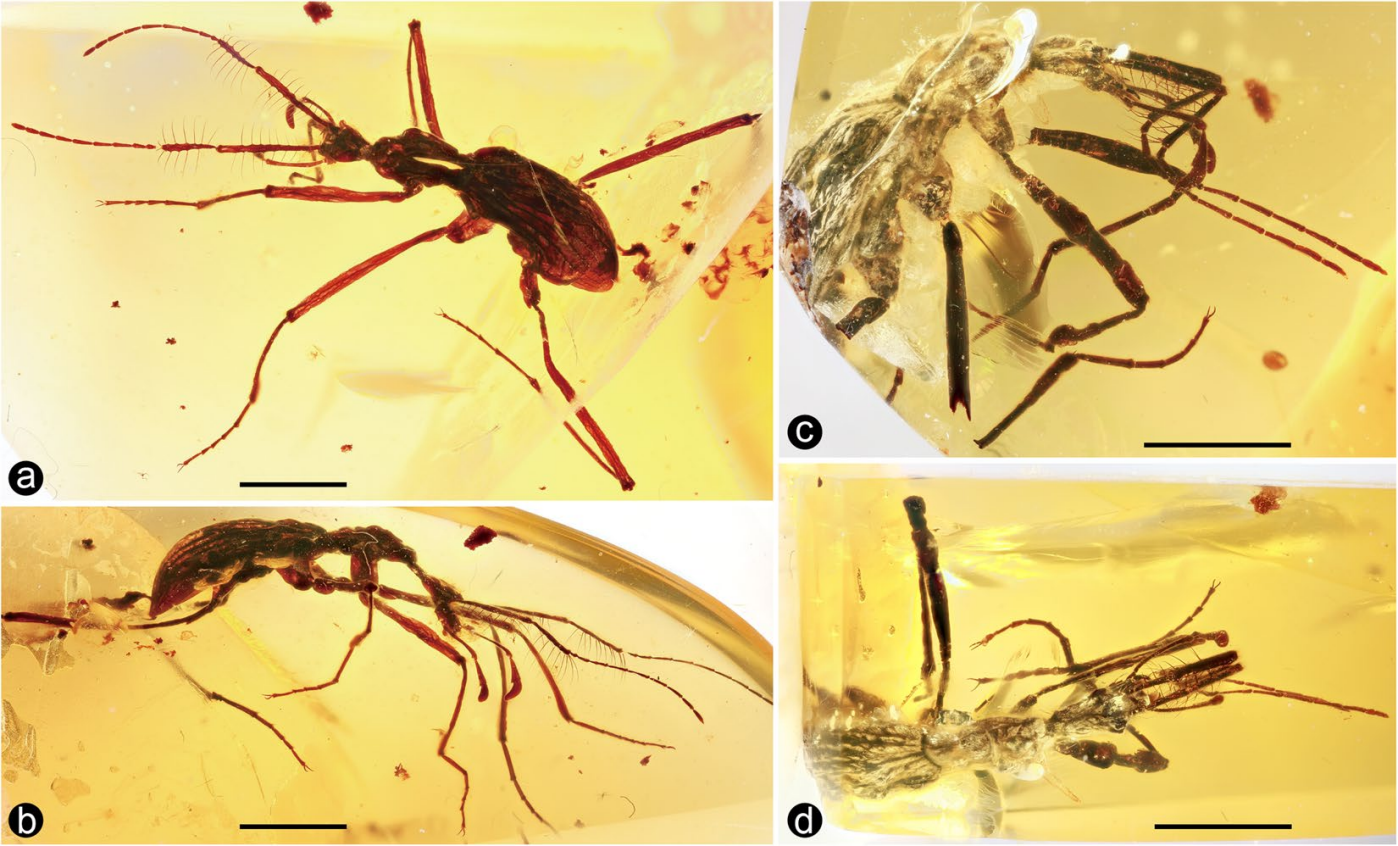
Habitus of *Cascomastigus monstrabilis* (**a**,**b**: male, SNUC-Paleo-0005; **c**,**d**: female, NIGP165026)**. (a)** Dorsolateral view. **(b**,**c)** Lateral view. **(d)** Dorsal view. Scale bars: 2 mm.

**Figure 2:**
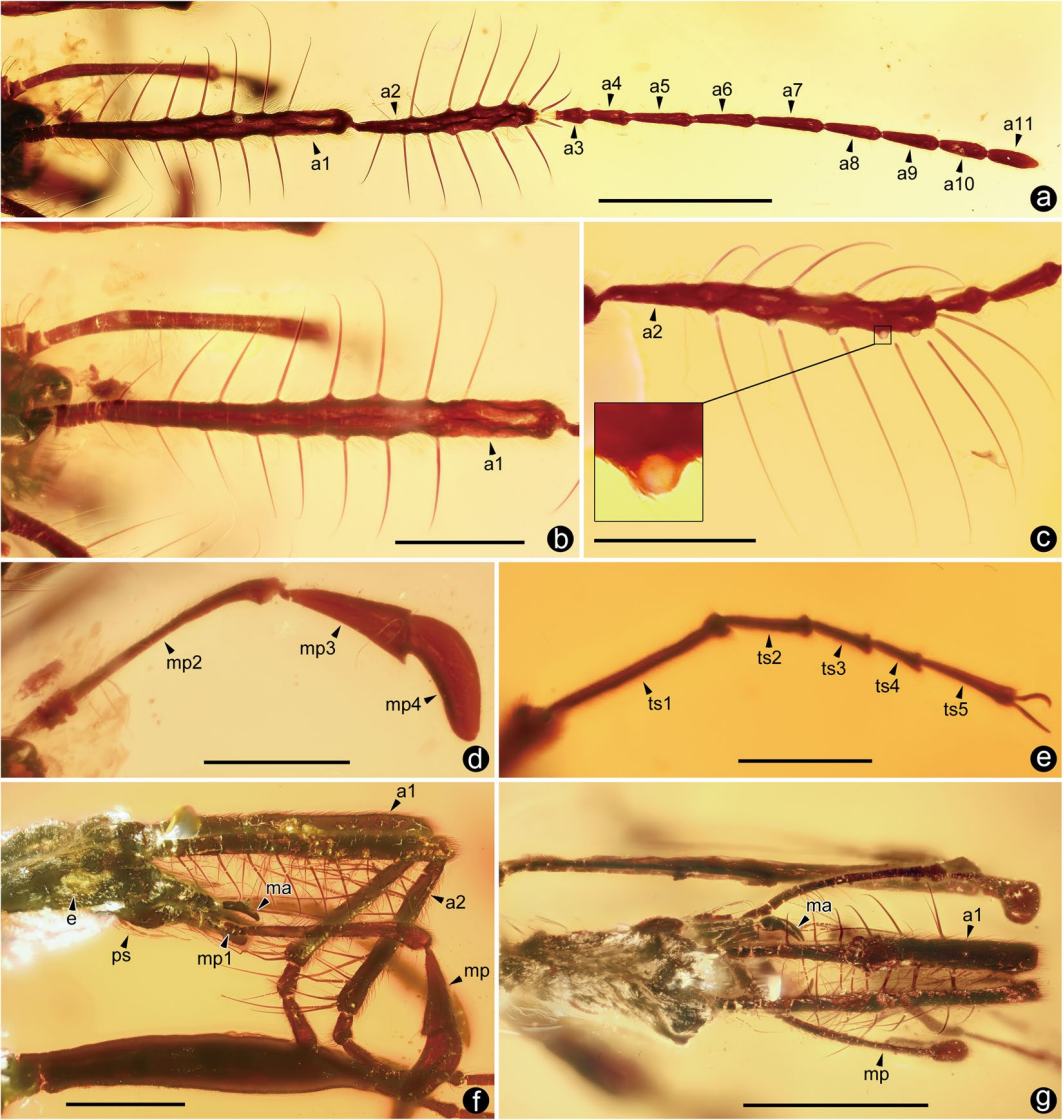
Morphological details of *Cascomastigus monstrabilis* (**a**–**e**: male; **f**,**g**: female). (**a)** Left antenna. **(b)** Left antennomere I. **(c)** Left antennomere II, and an enlarged image of a single basal socket. **(d)** Left maxillary palpus. **(e)** Right mesotarsomeres. **(f)** Head in lateral view, showing antennomeres I–II and maxillary palpi. **(g)** Same, in dorsal view. Abbreviations: a1–11, antennomeres I–XI; e, compound eye; ma, mandible; mp, maxillary palpus; mp1–4, maxillary palpomeres I–IV; ps, postgenal setae; ts1–5, tarsomeres I–V. Scale bars: 1 mm in A and E; 500 μm in others.

The three specimens presented here belong to two different species of a new genus — *Cascomastigus monstrabilis* (both sexes known; Fig. 1), and an unnamed species *Cascomastigus* (female). *Cascomastigus* are unambiguously referred to the extant ant-like stone beetle supertribe Mastigitae, a small group of Scydmaeninae (Staphylinidae), based on multiple putative synapomorphies: antennomere I (scape) elongate, much longer than antennomere II (pedicel) (Fig. 2a); antenna distinctly geniculate between scape and pedicel (Fig. 2f); maxillary palpus longer than head, with palpomere IV slightly longer than palpomere III and strongly asymmetrical (Fig. 2f); and compound eyes located in the anterior part of the head^7, 8^. Moreover, *Cascomastigus* are placed in the extant tribe Mastigini based on the presence of two longitudinal rows of robust spines on the basal two antennomeres (Fig. 2a), antennomere II broader than the flagellomeres (Fig. 2a), and the apical maxillary palpomere broader than palpomere III (Fig. 2d). The discovery of three definitive mastigine fossils extends the earliest records of the tribe by about 54 million years, the next oldest being those species in mid-Eocene Baltic amber (approximately 45 mya)^9^.

With a body length about 6.9 mm, these Cretaceous *Cascomastigus* are markedly larger than most extant Scydmaeninae (usually less than 2 mm long), and are indicative of Mastigitae, a group including the largest known Scydmaeninae (approximately 10 mm long). Like the modern mastigine genus *Stenomastigus*, the legs and antennae of *Cascomastigus* are extremely slender and elongate (Fig. 2a,b). The long legs, including tarsi (Fig. 2e), are probably suggestive of active and rapid locomotion. Like their modern counterparts, *Cascomastigus* likely moved quickly on substrates, likely the forest floor (soil or humus layer). The hind wings are not visible and the elytra are strongly constricted basally, tending to indicate that *Cascomastigus* were likely flightless, similar to many extant mastigines. Considering the extremely similar body form and greatly extended appendages to those of extant species, *Cascomastigus* were likely also diurnal predacious beetles, occurring in loose layers of leaf litter, and on the soil surface^10^. The elytra of *Cascomastigus* are distinctly striate, with each elytron bearing six longitudinal punctate striae (Supplementary Figs 1b and 2a,b). This feature is not known among extant Mastigini, but somewhat similar elytral punctures partly arranged in longitudinal rows can be found in the Eocene *Baltostigus* ^9^. *Cascomastigus* bear elongate maxillary palpi, with slightly enlarged and basally bent apical palpomeres (Fig. 2d). By contrast, the apical maxillary palpomeres of *Baltostigus* are strongly enlarged and axe-shaped^9^, a character more reminiscent of that of extant members of Mastigini, suggesting that the fossils may form a group sister to the remainder of the tribe. This is supported by the putatively primitive form of the elytra, along with the slightly enlarged apical maxillary palpomere in both groups. The mandibles of *Cascomastigus* have a sharp preapical tooth and apex (Fig. 2f,g), suggesting a predatory lifestyle, as in modern mastigine beetles.

The most remarkable feature of *Cascomastigus* is the densely spiny antennal scape and pedicel (Fig. 2a–c, f,g; Supplementary Fig. 2c), and similarly dense, strong and long bristles are only found in *Baltostigus* (Supplementary Fig. 3a)*.* The first antennomere (scape) is elongate and enlarged in all known Mastigitae, but it is armed with rows of bristles only in Mastigini. Compared to those of extant genera, the bristles on the scape and pedicel of *Cascomastigus* are much denser and larger (Fig. 2a–c). The scape of *Cascomastigus*, as long as the following four antennomeres combined, is armed with about 17 strong lateroventrally directed bristles arranged in two regular longitudinal rows (Fig. 2b). The long bristles on the scape gradually increase in length towards apex, with the apical bristles slightly shorter than the penultimate one (Fig. 2b). Similar to the scape, the pedicel is broad and elongate, about 6.5 times as long as the third antennomere (Fig. 2c). The pedicel bears about 13 lateroventrally directed bristles, which gradually shorten apically (Fig. 2c). By contrast, the following nine antennomeres are not morphologically specialized and devoid of such long bristles (Fig. 2a). All bristles are gradually tapered, with the greatest diameters at their bases (Fig. 2b,c). The bristles on the scape range from 0.26 mm to 0.81 mm in length, with average length about 0.46 mm distributed mainly in the median part. The bristles on the pedicel range from 0.25 mm to 0.62 mm in length, with average length about 0.48 mm. The bristles on scape and pedicel are largely of symmetrical, as evidenced by their similar lengths and relatively constant spacing (Fig. 2a), and more importantly, by the fact that the antennal scape bears a ventral notch, so that the pedicel can bend ventrally (Fig. 2f). Another interesting feature is that the most apical bristle on the scape and the most basal bristle on pedicel are somewhat of central symmetry (Fig. 2a), enabling these two bristles to form a functional setal pair when the antennae are folded. All of the lateroventrally directed bristles stand at right angles relative to the antennal length, so that bristles on the inner margins cross each other when the basal two antennomeres are brought together (Fig. 2g). When the antennae are folded, the scape and pedicel together form a well closed “cage” (Fig. 2f), representing a form of specialization perfectly analogous to that of the modern ground beetle genus *Loricera* (Carabidae: Loricerinae)^11, 12^. The sockets of these bristles are robust, with their openings facing lateroventrally (Fig. 2c), making the bristles unable to bend upwards. This structure is perfectly analogous to the functional, ventrally positioned setae on the basal antennomeres of species of *Loricera* ^12^. This antennal setal trap is used for preying upon springtails in *Loricera*, a commonly encountered hexapod living on the same damp, shady soil as these beetles^11, 12^. The presence of an antennal setal trap that corresponds so perfectly in structure to that found among *Loricera* strongly suggests that the similar trap in *Cascomastigus* was likely used for similar purposes.

Collembola are of considerable antiquity and include some of the earliest fossils of all hexapods. As noted, the earliest springtail, *R. praecursor* from the earliest Devonian, can be putatively attributed to an extant family, demonstrating a remarkable conservatism in their general morphology^3, 4^. Given their small sizes and soft bodies, the fossil record of Collembola is rather sparse, with only one other occurrence from the Paleozoic^13^. However, by the Cretaceous and the prevalence of amber deposits, ideally suited for the preservation of such minute arthropods, there is a diverse and rich record of springtails (summarized by Sánchez-García and Engel)^14^, and in some localities and pieces they can be numerous^15–19^. Burmese amber harbors one of the most diverse faunas of Collembola, represented by 14 described species in 13 genera^16^. Most Cretaceous collembolans are remarkably similar to their extant relatives, emphasizing the antiquity and considerable morphological stasis of the group^17^. Collembola would have been an abundant resource for predators in the Burmese amber environment, assuming they could be caught. *Cascomastigus*, with the aid of their specialized antennal setal trap, probably preyed on springtails occurring in the same habitat. Moreover, modern springtails often form large aggregations, and such aggregations are not uncommon in Burmese amber (Supplementary Fig. 3b). Like their extant counterparts, *Cascomastigus* probably moved quickly on the soil surface, enabling frequent encounters with prey such as springtails^10^. The antennae of *Cascomastigus* were likely largely stretched forward during movement, similar to the posture preserved in the holotype of *C. monstrabilis* (Fig. 1a,b). When stimulated by a potential prey, *C. monstrabilis* likely snapped the antennae together (Fig. 1c,d), bent the pedicel, and pushed its head against the ground. The prey would be caught in the trap formed by the strong bristles and then seized by the mouthparts.

Although all extant mastigines lack huge and strong bristles on the scape and the pedicel, the Eocene *Baltostigus* do possess bristles similar to those of *Cascomastigus* (Supplementary Fig. 3a)^9^. Thus, a more parsimonious explanation for the evolution of antennal bristles would be that the inconspicuous retention of small bristles on the scape and the pedicel of all extant taxa is secondarily derived, with the condition in the fossils plesiomorphic. On the other hand, it is also possible the presence of thickened bristles in *Cascomastigus* and *Baltostigus* are a derived condition, and became extinct in subsequent evolutionary events. Like the Cretaceous *Cascomastigus*, the Eocene *Baltostigus* were likely capable of trapping prey using the antennal bristles. There was apparently a shift of feeding habits (prey choice) during the evolutionary history of Mastigini: early mastigines, including *Cascomastigus* and *Baltostigus*, probably fed on fast-moving animals, presumably early springtails; whereas all extant mastigines (*Mastigus*, *Palaeostigus*, and *Stenomastigus*) feed on slow-moving organisms such as caterpillars or scavenge dead arthropods. Consequently, during the long evolutionary process, the scape and pedicel of extant mastigines might have reverted to a more typical function, no longer serving in prey capture, and becoming reduced in size.

**Figure 3:**
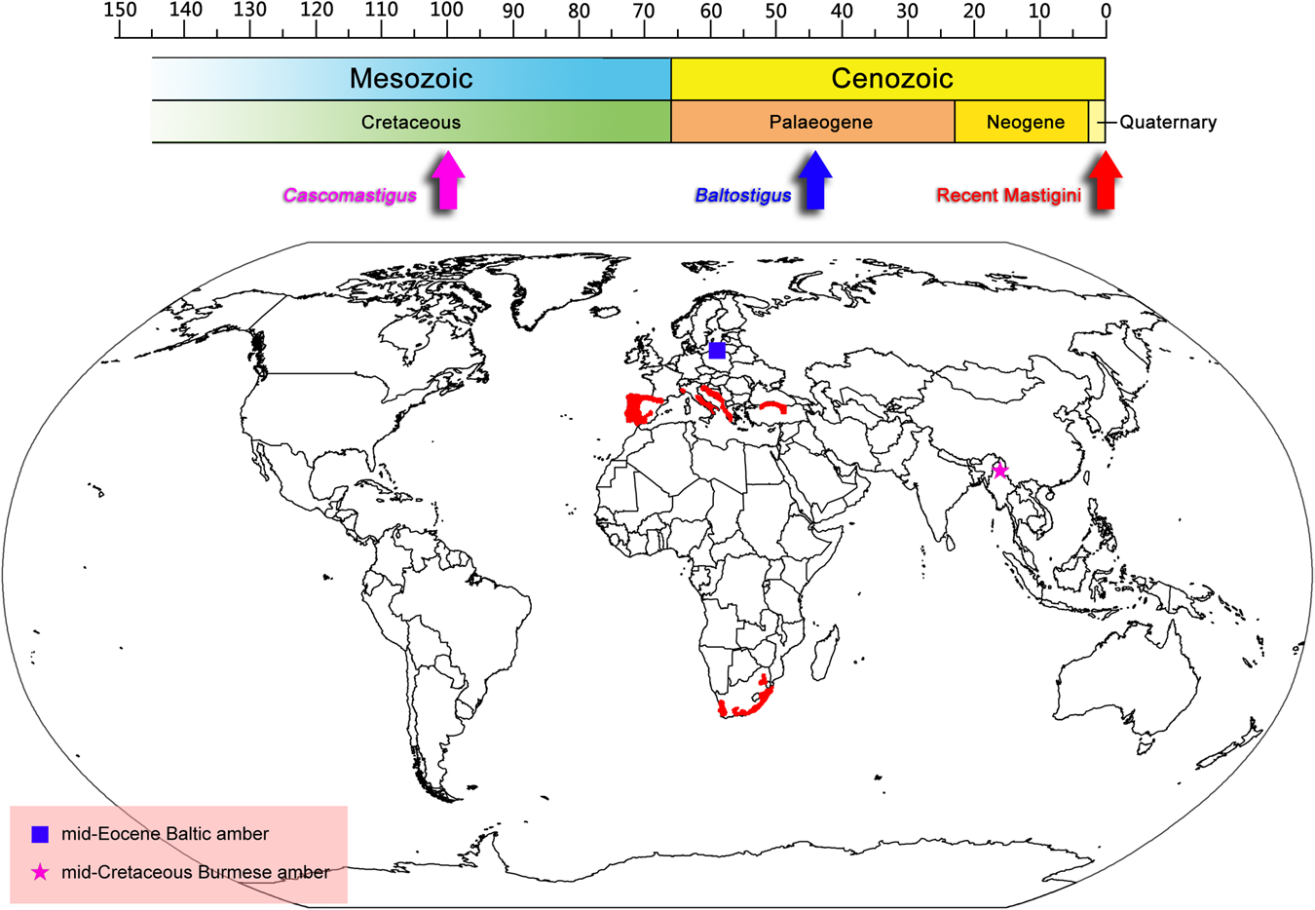
Spatiotemporal distribution of extant and amber-embedded Mastigini. Recent distribution of Mastigini is represented in red. Mastigini-yielding fossil localities are indicated in blue for *Baltostigus* (Baltic amber), and in pink for *Cascomastigus* (Burmese amber). The temporal distribution of fossil and extant Mastigini is indicated by arrows of the corresponding colors. (The original map source was obtained from http://www.simplemappr.net, an on-line tool for creating maps that can be freely used for publications and presentations).

The extant tribe Mastigini display a disjunctive distribution (Fig. 3)^10^. Two genera, *Mastigus* and *Stenomastigus*, occur only in South Africa, whereas *Palaeostigus* are known from southern Europe and South Africa^9^. It is apparent that the extant genera of Mastigini represent only a fraction of the group's true diversity^9^, and the lineage perhaps experienced significant extinction over the last 100 million years. Along with *Baltostigus* from Poland and Lithuania (Baltic amber), our discovery of *Cascomastigus* from the earliest Late Cretaceous amber of northern Myanmar further highlights the once broader distribution of Mastigini (Fig. 3). It also suggests that Mastigini appeared comparatively early, no later than the mid-Cretaceous (approximately 99 mya). Interestingly, Burmese amber harbors a relative rich fauna of scydmaenines^20^, although none of those had such antennal modifications. The mastigine forms with a functional antennal setal trap appear to have persisted for at least 54 million years.

**Figure 4:**
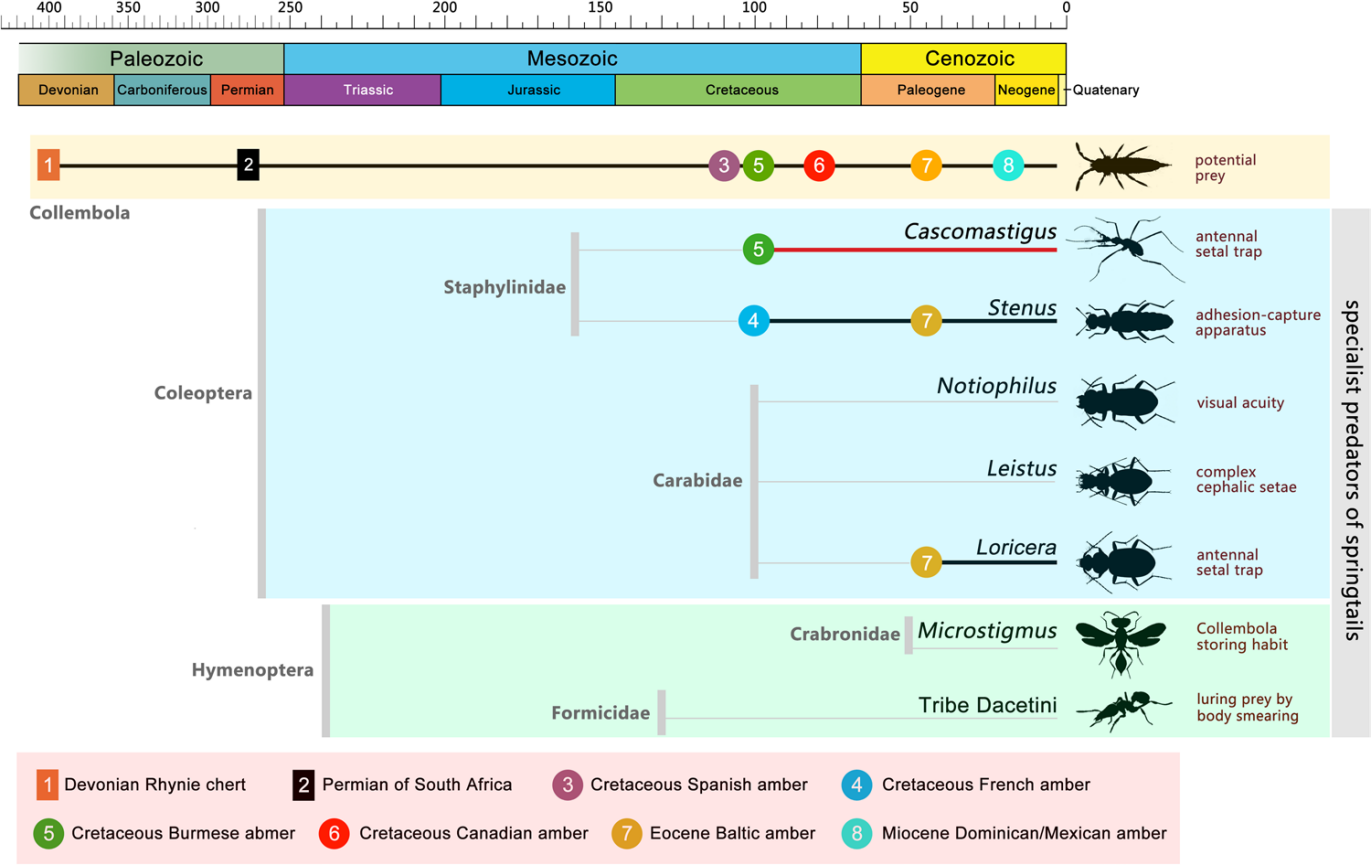
Time-dated chronogram of specialist predators of Collembola in various insect groups. Their respective predatory strategies are provided at the right.

Despite their abundance, obligate predators of springtails are of low diversity^6^. Among the known specialized collembolan predators, various ground-dwelling beetles adopt different preying strategies for capturing springtails (Fig. 4). Such beetles belong to the Staphylinidae (rove beetles: some *Stenus*) and Carabidae (ground beetles: *Leistus*, *Loricera*, and *Notiophilus*). Specifically, *Stenus* catch elusive prey using a specialized adhesive prey-capture apparatus, which is modified from the labium^21, 22^. *Leistus* possess a hunting apparatus formed of strong setae in a circular arrangement on the ventral head surface^23^. Adults of *Loricera* have the aforementioned antennal setal trap^11, 12^; while larvae have elongate and strong maxillae with a long and tacky galea for hunting springtails^24^. *Notiophilus* hunt their prey based on visual acuity^25^. In addition, unusual adaptions for collembolan predation are also found in some wasps and dacetine ants^6^. Therefore, it is apparent that different morphological modifications for catching Collembola have originated independently for multiple times among insects. There are records of *Stenus* and *Loricera* in Baltic amber^26–29^, but only *Stenus archetypus* preserves an exposed adhesive prey-capture apparatus^29^. Two *Stenus* are recorded from the Cretaceous, but these are insufficiently preserved and specialized mouthparts, if they occurred, are unknown^26, 27^. A specialized larva of *Loricera electrica* with strong maxillae and bulb-shaped galeae has been reported from Baltic amber^28^, and an undescribed adult of *Loricera* with a typical antennal setal trap has also been recovered from the same deposits (Supplementary Fig. 3c,d). Thus, all previously known specialist predators of springtails are confined to the middle Eocene. The discovery of *Cascomastigus* from the mid-Cretaceous represents the earliest known predators specialized for capturing Collembola, pushing back the age of such predation by at least 54 million years.

## Methods

All amber pieces were cut by a handheld engraving tool with a diamond blade, then ground with emery papers of different grain sizes, and finally polished with polishing powder. Photographs were taken using a Canon EOS 5D Mark III digital camera, equipped with a Canon MP-E 65 mm macro lens (F2.8, 1–5X), and with an attached Canon MT-24EX twin flash. Focus stacking software (Zerene Stacker, Version 1.04) was used to increase depth of field. All images were modified and arranged in Adobe Photoshop CS5 Extended.

## Electronic supplementary material

Supplementary Information
