# Supplementary material for: Specialized adaptations for springtail predation in Mesozoic beetles
Source: Sci Rep. 2017 Mar 7;7:98. doi: 10.1038/s41598-017-00187-8 (PMC5427802; doi:10.1038/s41598-017-00187-8)
Supplement: Supplementary file 1 — Supplementary Information [file 41598_2017_187_MOESM1_ESM.pdf]

**SUPPLEMENTARY INFORMATION**

**Title: Specialized adaptations for springtail predation in Mesozoic beetles**

**Authors:** Zi-Wei Yin, Chen-Yang Cai, Di-Ying Huang & Li-Zhen Li

**Systematic Palaeontology**

Order Coleoptera Linnaeus, 1758

Family Staphylinidae Latreille, 1802

Subfamily Scydmaeninae Leach, 1815

Supertribe Mastigitae Fleming, 1821

Tribe Mastigini Fleming, 1821

***Cascomastigus* Yin & Cai, gen. nov.**

LSID urn:lsid:zoobank.org:act:08FE9CB6-4F93-4F68-B397-F2B6D392AF36

**Type species.** *Cascomastigus monstrabilis* Yin & Cai, sp. nov.

**Diagnosis.** *Cascomastigus* are separated from other genera of the Mastigini by the following combination of characters: body size exceptionally large (usually over 6.5 mm); maxillary palpus extremely elongate, with enlarged apical palpomere (palpomere IV) strongly bent basally; and elytron distinctly striate.

**Etymology.** The generic name is composed of the Latin '*cascus*' meaning 'ancient, primitive', and '*Mastigus*', the type genus of Mastigini. The gender is masculine.

**Systematic placement of *Cascomastigus*.** *Cascomastigus* are readily assigned to the supertribe Mastigitae based on the presence of several putative synapomorphies of the group: 1) antennomere I (scape) elongate, much longer than antennomere II (pedicel); 2) antenna distinctly geniculate between scape and pedicel, so that the pedicel and flagellomeres are able to bend ventrally; 3) maxillary palpus longer than head, with palpomere IV slightly longer than palpomere III and strongly asymmetrical; and 4) compound eyes located in the anterior part of the head[1–3]. Within Mastigitae three tribes are currently established[4]: Leptomastacini, Mastigini, and Clidicini. *Cascomastigus* undoubtedly belong to the tribe Mastigini on the basis of the two longitudinal rows of specialized macrosetae along the ventral

margins of the scape and pedicel; enlarged pedicel compared to flagellomeres; and maxillary palpomere IV broader than III. Within Mastigini, three Recent (*Mastigus* Latreille, *Stenomastigus* Leleup, *Palaeostigus* Newton) and one extinct (*Baltostigus* Jałoszyński) genera have been described. *Cascomastigus* can be readily separated from all Recent genera by the maxillary palpomere IV being strongly bent basally, and the distinctly striate elytra, whereas all three Recent genera have relatively straight and elongate maxillary palpomere IV, and the elytra are impunctate and lack any striae. The recently described *Baltostigus* from Eocene Baltic amber possesses a relatively much more convex body, the body size is less than 3.5 mm, and the maxillary palpus IV is axe-shaped and distinctly broader than long, and therefore can be easily distinguished from *Cascomastigus*. It is noteworthy that *Baltostigus* possess fully-developed hind wings and strongly-developed humeral calli on the elytra, which were considered as primitive relative to all Recent Mastigini[5]. In *Cascomastigus* the body and its appendages are extremely elongate, and the elytra are strongly constricted basally. Such morphology indicates that species of *Cascomastigus* are most likely flightless, and instead they are likely capable of rapid movement in open habitats, similar to many extant mastigine species. Given the presence of a few aforementioned primitive character states and the age of the new taxon (earliest Cenomanian), we therefore posit *Cascomastigus* as a relative of *Baltostigus*, although this requires testing by future cladistic analyses.

***Cascomastigus monstrabilis* Yin & Cai, sp. nov.**

LSID urn:lsid:zoobank.org:act:7D363AB7-A803-4B30-A375-7AF3B46E88CF

**Type material.** Holotype, SNUC-Paleo-0005, a completely preserved male; Cretaceous Burmese amber, lower Cenomanian, near Tanai, Hukawng Valley, Kachin State, Myanmar; housed in the Insect Collection of the Shanghai Normal University, Shanghai, China. Paratype, NIGP165026, presumably a female, with posterior half of elytra, abdomen, right middle leg, and hind legs not preserved; occurrence and locality data same as for the holotype; housed in the Nanjing Institute of Geology and Palaeontology, CAS, Nanjing, Jiangsu Province, China.

**Etymology.** The specific epithet '*monstrabilis*' refers to the horrible appearance of the beetle with exaggerated bristles on the first two antennomeres.

**Locality and age.** Derived from an amber mine located near Noije Bum, Tanaing, Kachin, Myanmar. The U–Pb dating of zircons from the volcanoclastic matrix yielded an age of 98.79 ± 0.62 million years.

**Description.** Body (Fig. 1) elongate and uniformly reddish-brown, including vestiture; body length 6.88 mm (male). Head (Figs 1B, 1D, 2G) much longer than broad, broadest at compound eyes, length (including occipital constriction) 1.20 (male)–1.42 (female) mm, width 0.78 mm; occipital constriction deep and distinct, postocular margins narrowing posteriorly, more than twice as long as compound eye diameter; compound eyes prominent, slightly oval and strongly convex, finely faceted, vertex slightly impressed medially; frons in lateral view forms a distinctly obtuse angle with vertexal plane, its posterior margin delimited by antennal sockets, lacking median longitudinal ridge between antennae, median part of frons slightly impressed and covered with sparse, short, erect setae; maxillary palpus with minute and weakly elongate palpomere I (Fig. 2F), strongly elongate and slender palpomere II, subtriangular and elongate palpomere III broadest at apex, and broad palpomere IV strongly bent basally; measurements of palpomeres II–IV (female in parentheses): 1.05 (1.11) mm, 0.48 (0.48) mm, 0.50 (0.51) mm; labial palpus not clearly visible in available specimens. Antenna (Fig. 2A) slightly shorter than body, length 5.94 (male)–6.49 (female) mm, lengths of antennomeres (female in parentheses): I 1.94 (1.85) mm, II 1.13 (1.04) mm, III 0.19 (0.19) mm, IV 0.24 (0.33) mm, V 0.37 (0.39) mm, VI 0.35 (0.50) mm, VII 0.41 (0.48) mm, VIII 0.35 (0.48) mm, IX 0.35 (0.46) mm, X 0.30 (0.39) mm, XI 0.31 (0.39) mm; relative lengths of antennomeres I–XI (female in parentheses): 1.0 (1.0) : 0.58 (0.56) : 0.1 (0.1) : 0.12 (0.18) : 0.19 (0.21) : 0.18 (0.27) : 0.21 (0.26) : 0.18 (0.26) : 0.18 (0.25) : 0.15 (0.21) : 0.16 (0.21); scape strongly elongate and slightly broadening distally, 1.30 (female)–1.62 (male) times as long as head, 0.96 (female)–1.13 (male) times as long as pronotum, and about as long as half elytral length in male, with two lateroventral rows of robust bristles (7 in mesal row and 8 in lateral row); pedicel 0.56 (female)–0.58 (male) times as long as scape, also with two lateroventral rows of long bristles (7 in mesal row and 6 in lateral row); all flagellomeres strongly elongate, with dense, short and suberect setae. Pronotum (Supplementary Fig. 1A) elongate and broadest near anterior third, length 1.72 (male) –1.92 (female) mm, width 0.98 (male) mm; anterior margin arcuate, posterior margin truncate, probably lacking dorsal grooves or pits. Prothoracic hypomera not demarcated laterally; details of prosternum not observable. Elytra (Fig. 1B, Supplementary Fig. 1A–B) much more convex than pronotum, with highest and broadest point located distinctly behind middle, length 3.96 mm, width approximately 1.7 mm; each elytron lacking humerus, with six longitudinal striate rows; vestiture composed of sparse, short and suberect setae directed posteriorly. Hind wings not visible, putatively absent. Details of mesoventrite not visible, except presence of prominent mesocoxal projection (Supplementary Fig. 1C). Metaventrite not clearly observable. Abdomen about as long as metaventrite; abdominal sternites III–VIII clearly visible (Supplementary Fig. 1D), sternite III about

same length as sternites IV–V combined, sternites III–VI subequal in length, sternite VII about 1.5 times longer than sternite VI, sternite VIII twice as long as VII, roundly triangular, posterior margin deeply emarginate medially (Supplementary Fig. 1D, indicated by dotted line), apical portion of sternite IX exposed, with rounded posterior margin. Legs (Fig. 1) long and slender; large coxae subconical; trochanters lengthy semi-rounded, trochantero-femoral junction strongly oblique; femora indistinctly clavate, covered with dense, short and nearly recumbent setae; all tibiae slender, slightly curved, shorter than corresponding femora, densely covered with short and nearly recumbent setae; lengths of femora and tibiae (female in parentheses): pro-femur/tibia 1.24 (1.41)/0.89 (1.11) mm, meso-femur/tibia 1.68 (1.57)/1.24 (1.41) mm, meta-femur/tibia 1.87/1.42 mm; each tarsus distinctly longer than half length of corresponding tibia, all tarsomeres distinctly cylindrical, slightly broadened apically, tarsomere I longest, with two symmetrical, curved and pointed claws. Aedeagus not visible.
**Comments.** The holotype is assuredly male base on the deeply emarginate posterior margin of abdominal sternite VIII. Though the posterior part of the body is not preserved, the paratype seems to be distinctly larger than the holotype (inferred by the combined length of head + pronotum). In Mastigini, females are often much larger than males, having a different elytral shape and possessing shorter antennae in relation to the whole body length[5–8]. The paratype is tentatively assigned as a female in association with the holotype male given its larger size, but the poor preservation of this specimen renders further assessment of its gender difficult.

***Cascomastigus* sp.**

**Material examined.** SNUC-Paleo-0006, a partially preserved female; Cretaceous Burmese amber, lower Cenomanian, near Tanai, Hukawng Valley, Kachin State, Myanmar; housed in the Insect Collection of Shanghai Normal University, Shanghai, China.
**Measurements.** lengths: 5.74 mm (body), 1.01 mm (head), 1.52 mm (pronotum), 2.53 mm (head + pronotum), 3.21 mm (elytra), 1.55 mm (scape), 1.0 mm (pedicel); widths: 0.84 mm (head), 1.07 mm (pronotum), 1.70 mm (elytra); scape length / head length = 1.53, scape length / pronotal length = 1.02; elytral length / scape length = 2.07.
**Comments.** The general morphology (Supplementary Fig. 2A–D) of this female is similar to *C. monstrabilis*, but due to the smaller body size and different proportions of the body parts, it probably represents a different species. Pending the discovery of an associated male, this specimen is not formally named here.

**Supplementary references**


1. O'Keefe, S. T., Pike, T. & Poinar, G. *Palaeoleptochromus schaufussi* (gen. n., sp. nov.), a new antlike stone beetle (Coleoptera: Scydmaenidae) from Canadian Cretaceous amber. *Can. Entomol.* **129,** 379–385 (1997).
2. O'Keefe, S. T. Scydmaenidae Leach, 1815. In Handbook of Zoology, Coleoptera, Beetles, v. 1: Morphology and systematics (Archostemata, Adephaga, Myxophaga, Polyphaga partim) (eds Beutel, R. G. & Leschen, R. A.) 280–288 (De Gruyter, New York, 2005).
3. Jałoszyński, P. Description of *Euroleptochromus* gen. n. (Coleoptera, Staphylinidae, Scydmaeninae) from Baltic amber, with discussion of biogeography and mouthpart evolution within Clidicini. *Syst. Entomol.* **37,** 346–359 (2012).
4. Newton, A. F. & Franz, H. World catalog of the genera of Scydmaenidae (Coleoptera). *Koleopterol. Rundsch.* **68,** 137–165 (1998).
5. Jałoszyński, P. A new Eocene genus of ant-like stone beetles sheds new light on the evolution of Mastigini. *J. Paleontol.* **89,** 1056–1067 (2015).
6. Jałoszyński, P. South African *Stenomastigus* Leleup (Staphylinidae, Scydmaeninae): status of subgenus *Acanthostigus* Leleup and revision of species with elongated protrochanters in males. *Zootaxa* **3153,** 39–56 (2012).
7. Jałoszyński, P. New synonymy and redescription of *Mastigus deustus* (Thunberg) (Coleoptera, Staphylinidae, Scydmaeninae). *Zootaxa* **3482,** 68–76 (2012).
8. Jałoszyński, P. *Stenomastigus pseudofranzi* sp. n., from South Africa (Staphylinidae, Scydmaeninae). *Zootaxa* **3268,** 55–62 (2012).

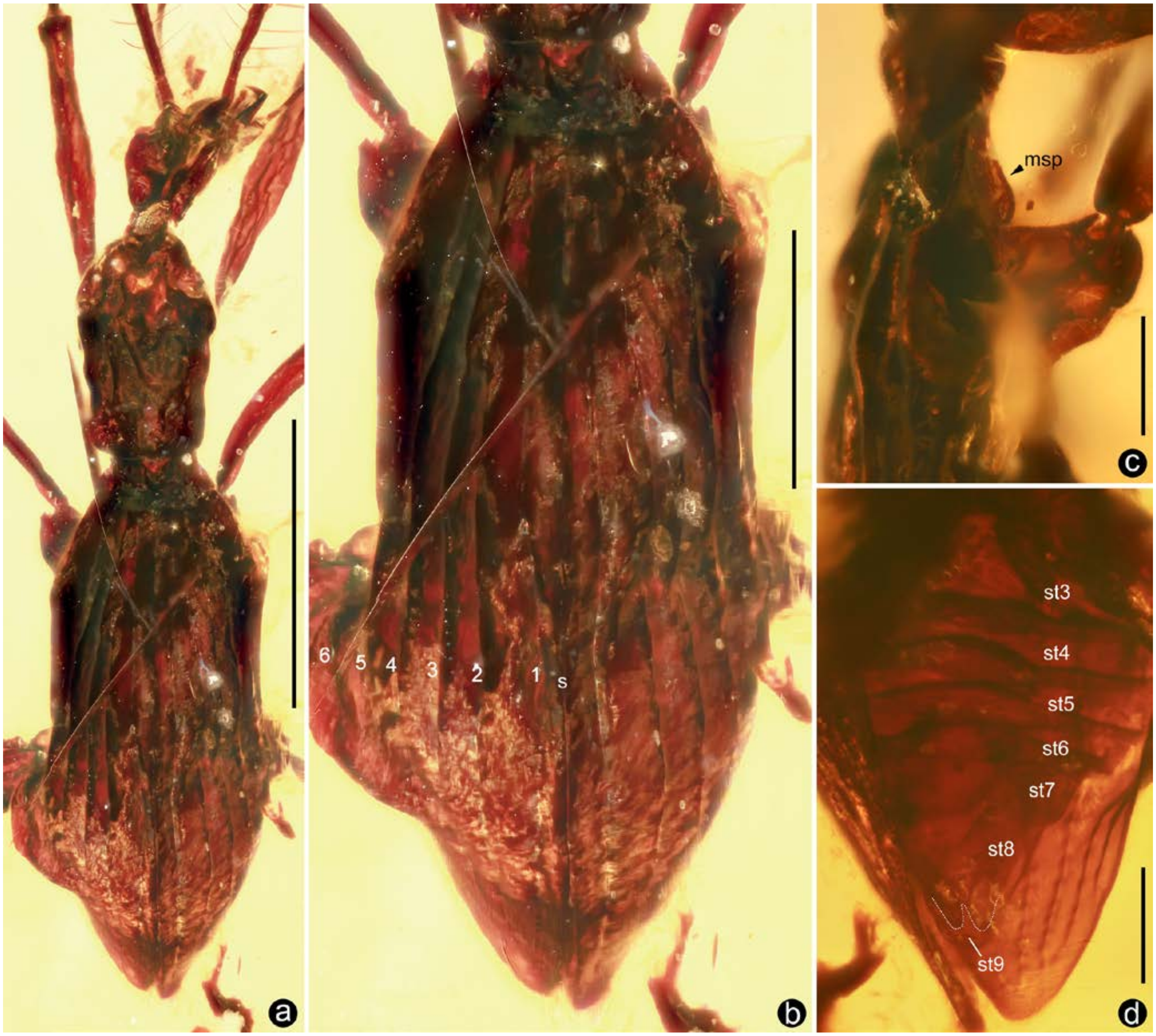


**Supplementary Figure 1. Morphological details of male *Cascomastigus monstrabilis*, SNUC-Paleo-0005. (a)** Dorsal view of head, pronotum, and elytra. **(b)** Elytra, enlarged. **(c)** Lateral view of meso-thorax, showing prominent mesocoxal projection. **(d)** Abdomen, in ventral view; dotted line indicates posterior margin of sternite 8. Abbreviations: 1–6, elytral longitudinal striae 1–6; msp, mesocoxal process; s, suture; st3–9, sternites 3–9. Scale bars: 2 mm in a; 1 mm in b–d.

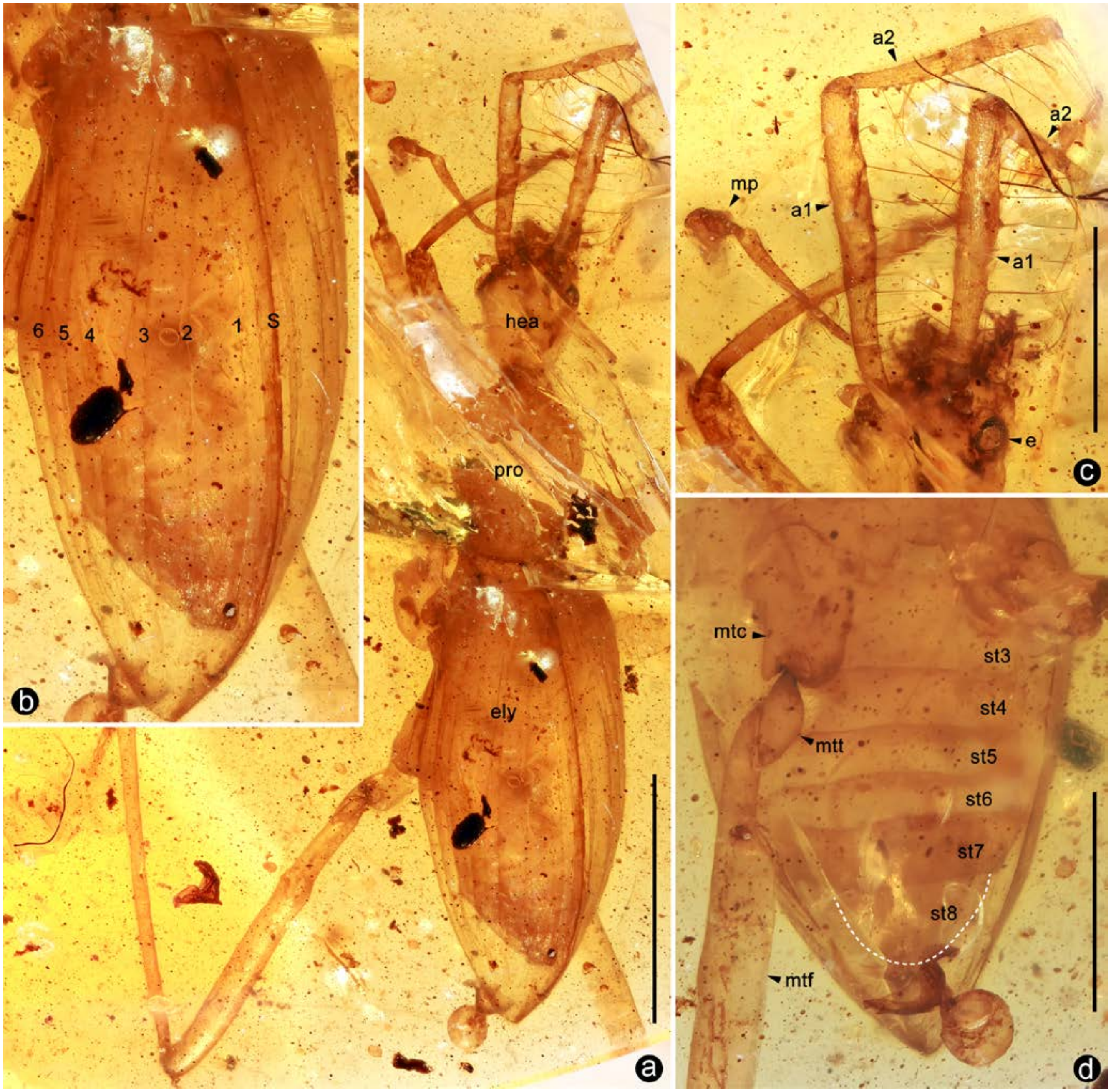


**Supplementary Figure 2. Morphological details of *Cascomastigus* sp. female, SNUC-Paleo-0006.** **(a)** Dorsal habitus. **(b)** Elytra, enlarged. **(c)** Appendages of the head. **(d)** Abdomen, in ventral view; dotted line indicates posterior margin of sternite 8. Abbreviations: 1–6, elytral longitudinal striae 1–6; a1–2, antennomeres 1–2; e, eye; ely, elytra; hea, head; mp, maxillary palpus; mtc, metatrochanter; mtf, metafemur; mtt, metatrochanter; pro, pronotum; s, suture; st3–8, sternites 3–8. Scale bars: 2 mm in a; 1 mm in c–d.

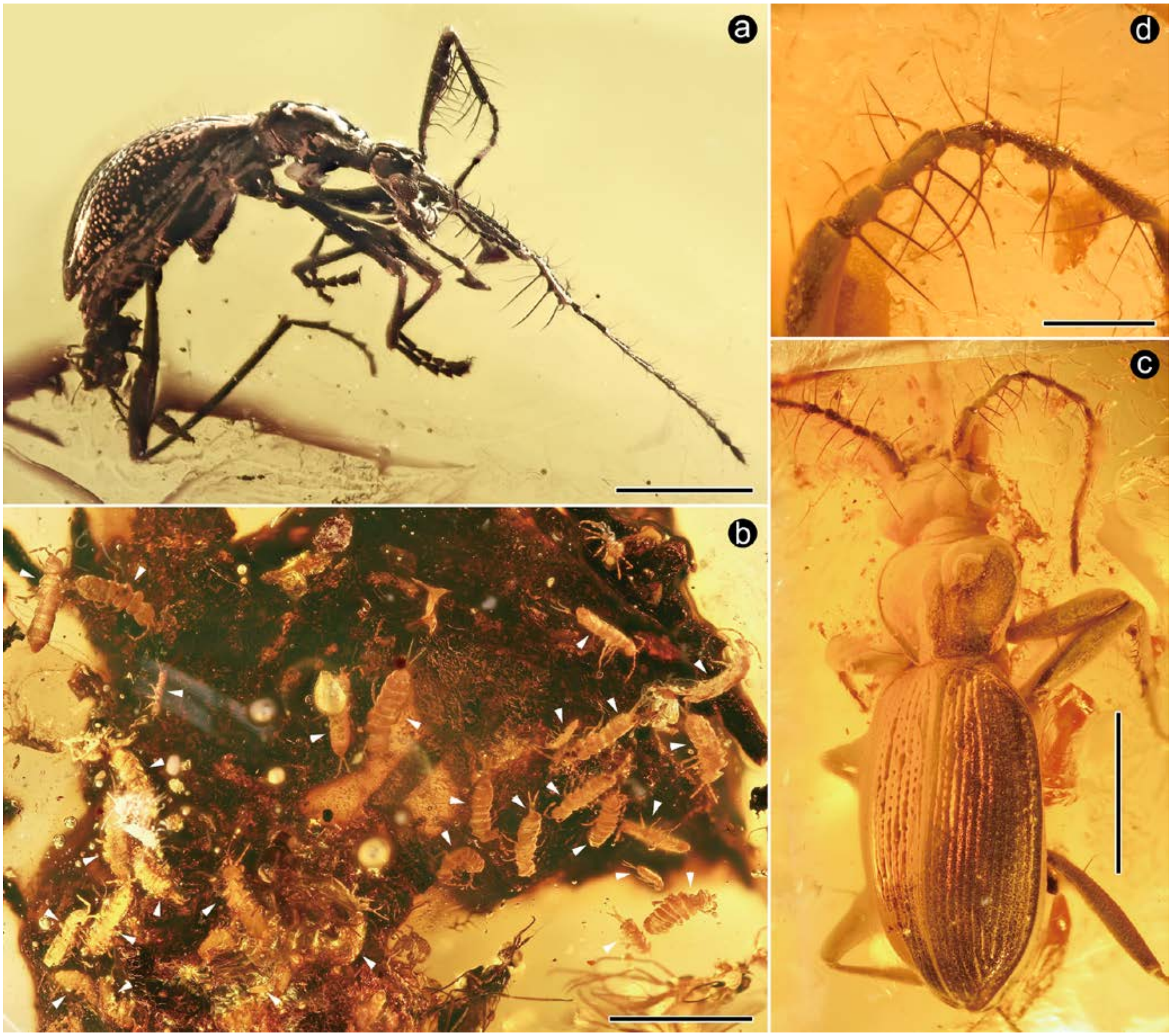


**Supplementary Figure 3. Comparative morphology of Eocene *Baltostigus* and *Loricera*, and potential food resource of *Cascomastigus*. (a)** *Baltostigus* sp. known from the middle Eocene Baltic amber. **(b)** Aggregations of Collembola in mid-Cretaceous Burmese amber, presumably as potential prey of *Cascomastigus*. **(c)** An undescribed *Loricera* sp. from the Baltic amber, showing **(d)** similar antennal structure for prey-capture. Scale bars: 1 mm in a; 2 mm in b–c; 0.5 mm in d.
